# Supplementary material for: Age-dependent changes in circulating Tfh cells influence development of functional malaria antibodies in children
Source: Nat Commun. 2022 Jul 18;13:4159. doi: 10.1038/s41467-022-31880-6 (PMC9293980; doi:10.1038/s41467-022-31880-6)
Supplement: Supplementary file 1 — Supplementary Material [file 41467_2022_31880_MOESM1_ESM.pdf]

Supplementary Material

Supplementary Table S1: Cohort clinical characteristics

	Cohort	
**Characteristics**	**Full ***	**Complete ***
Number	262	212
Age years (median, [IQR])	5.86 [3.7 - 8.3]	6.22 [3.8-8.6]
Female (n, %)	117 (45%)	96 (45%)
Infection at blood draw (n, %)	106 (40%)	89 (42%)
Household mosquite exposure (n, %)		
0-8	21 (8%)	19 (9%)
>8-40	124 (47%)	97 (46%)
>40-80	91 (35%)	77 (36%)
>80	26 (10%)	19 (9%)
Any infection in year following	241 (92%)	195 (92%)

* **Full cohort** - samples tested for antibody responses
Complete cohort - samples tested for both antibody and Tfh cell responses

Supplementary Material

Supplementary Table S2: Linear regression modelling outputs of the relationship of ICOS and Ki67 expression on Tfh subsets with age, *Pf* infection and household mosquito exposure

		univariate				adjusted *			
	**ICOS+**	**coef**	**95% CI**		**p**	**coef**	**95% CI**		**p**
Th1	age	-1.367	-1.872	-0.861	**<0.001**	-1.373	-1.900	-0.845	**<0.001**
	Pf infection	-1.432	-4.440	1.576	0.349	0.497	-2.433	3.426	0.739
	HME 0-8 (ref)								
	>8-40	0.456	-4.893	5.806	0.867	-1.229	-6.316	3.858	0.634
	>40-80	2.119	-3.343	7.582	0.445	0.123	-5.085	5.331	0.963
	>80	8.191	1.273	15.109	**0.021**	6.108	-0.474	12.690	0.069
Th2	age	-0.679	-1.089	-0.269	**0.001**	-0.644	-1.076	-0.212	**0.004**
	Pf infection	-1.116	-3.461	1.228	0.349	-0.187	-2.585	2.211	0.878
	HME 0-8 (ref)								
	>8-40	0.658	-3.550	4.866	0.758	-0.159	-4.324	4.006	0.940
	>40-80	2.205	-2.091	6.502	0.313	1.233	-3.031	5.497	0.569
	>80	4.714	-0.728	10.156	0.089	3.759	-1.630	9.148	0.171
Th17	age	-0.680	-1.137	-0.222	**0.004**	-0.553	-1.034	-0.073	**0.024**
	Pf infection	-2.719	-5.307	-0.132	0.349	-1.981	-4.651	0.689	0.145
	HME 0-8 (ref)								
	>8-40	1.548	-3.134	6.231	0.515	0.705	-3.928	5.339	0.764
	>40-80	2.217	-2.560	6.994	0.361	1.229	-3.510	5.967	0.610
	>80	5.462	-0.588	11.512	0.077	4.737	-1.251	10.725	0.120
	**Ki67+**	**coef**		**95% CI**	**p**	**coef**		**95% CI**	**p**
Th1	age	-0.362	-0.542	-0.182	**<0.001**	-0.436	-0.620	-0.251	**<0.001**
	Pf infection	0.883	-0.155	1.920	0.095	1.475	0.452	2.499	**0.005**
	HME 0-8 (ref)								
	>8-40	0.510	-1.345	2.365	0.589	0.055	-1.722	1.832	0.951
	>40-80	0.092	-1.802	1.986	0.923	-0.431	-2.250	1.389	0.641
	>80	2.790	0.391	5.189	**0.023**	2.059	-0.240	4.358	0.079
Th2	age	-0.080	-0.212	0.052	0.234	-0.119	-0.256	0.019	0.091
	Pf infection	0.686	-0.051	1.422	0.095	0.838	0.073	1.603	**0.032**
	HME 0-8 (ref)								
	>8-40	0.652	-0.678	1.983	0.335	0.555	-0.773	1.884	0.411
	>40-80	0.134	-1.225	1.492	0.846	0.028	-1.332	1.388	0.968
	>80	1.379	-0.342	3.100	0.116	1.157	-0.562	2.876	0.186
Th17	age	-0.248	-0.457	-0.040	**0.020**	-0.268	-0.488	-0.048	**0.017**
	Pf infection	0.048	-1.132	1.228	0.095	0.451	-0.769	1.670	0.467
	HME 0-8 (ref)								
	>8-40	1.217	-0.901	3.335	0.259	0.904	-1.213	3.020	0.401
	>40-80	0.308	-1.853	2.469	0.779	-0.052	-2.217	2.113	0.962
	>80	1.219	-1.518	3.956	0.381	0.793	-1.942	3.529	0.568

P values are two-sided and not adjusted for multiple comparisons

* There was no evidence for an interaction between age and infection on ICOS or Ki67 expression within the linear model.

Supplementary Material

Supplementary Table S3: Relationship between Tfh subsets and odds of infection

	Univariate				Adjusted			
**Tfh subset**	**OR**	**p**	**95% CI**		**aOR**	**p**	**95% CI**	
Tfh (% of CD4)	1.11	0.139	0.967	1.275	1.006	0.938	0.873	1.158
FoxP3 (% of Tfh)	0.908	0.475	0.697	1.183	0.94	0.62	0.737	1.199
Th1 (% of Tfh)	1.015	0.14	0.995	1.035	1.002	0.844	0.983	1.021
Th2 (% of Tfh)	0.996	0.644	0.979	1.013	1.01	0.261	0.993	1.027
Th17 (% of Tfh	0.946	**0.031**	0.9	0.995	0.943	**0.012**	0.9	0.987
ICOS (% of Tfh)	0.975	0.137	0.944	1.008	0.996	0.808	0.965	1.028
ICOS (% of Th1-Tfh)	0.992	0.468	0.971	1.014	1.006	0.621	0.984	1.028
ICOS (% of Th2-Tfh)	0.994	0.697	0.967	1.023	1.008	0.579	0.981	1.036
ICOS (% of Th17-Tfh)	0.986	0.256	0.963	1.01	1.002	0.899	0.978	1.025
Ki67 (% of Tfh)	1.012	0.803	0.924	1.107	1.013	0.772	0.927	1.108
Ki67 (% of Th1-Tfh)	0.99	0.765	0.93	1.055	0.995	0.874	0.935	1.059
Ki67 (% of Th2-Tfh)	1.051	0.32	0.953	1.158	1.054	0.277	0.958	1.16

N=211 children. OR: odds ratio; aOR: adjusted OR.

P values are two-sided and not adjusted for multiple comparisons

* Adjusted for age as a continuous variable, current *P. falciparum* infection detected by PCR, and household mosquito exposure as a categorical variable, 0-8, >8-40, >40-80, >80 mosquitos/household/day

Supplementary Material

Supplementary Table S4: Relationship between antibodies and odds of Infection

			**Univariate**			**Adjusted ***			
**Target**	**Type**	**OR**	**p**	**95% CI**		**aOR**	**p**	**95% CI**	
MSP2	IgG1	1.266	0.073	0.978	1.638	1.235	0.091	0.968	1.575
	IgG3	2.087	**<0.001**	1.648	2.643	1.941	**<0.001**	1.472	2.558
	IgM	1.202	0.222	0.895	1.613	0.992	0.958	0.743	1.326
	C1q	1.736	**<0.001**	1.292	2.331	1.565	**0.003**	1.168	2.097
	FcRIIa	1.568	**0.001**	1.221	2.013	1.380	**0.012**	1.074	1.773
	FcRIII	1.532	**<0.001**	1.226	1.916	1.354	**0.009**	1.079	1.701
	OPA	1.057	**<0.001**	1.036	1.079	1.044	**<0.001**	1.023	1.065
AMA1	IgG1	1.498	**<0.001**	1.286	1.745	1.349	**<0.001**	1.143	1.592
	IgG3	1.450	**0.036**	1.024	2.053	1.262	0.185	0.895	1.780
	IgM	1.605	**0.027**	1.055	2.441	1.101	0.666	0.712	1.703
	C1q	1.427	**0.009**	1.092	1.866	1.231	0.131	0.940	1.612
	FcRIIa	1.412	**<0.001**	1.216	1.640	1.294	**0.002**	1.101	1.519
	FcRIII	1.480	**<0.001**	1.225	1.788	1.313	**0.009**	1.071	1.609
	OPA	1.023	**<0.001**	1.012	1.035	1.013	**0.037**	1.001	1.025
CSP	IgG1	1.338	0.110	0.936	1.914	1.080	0.671	0.758	1.539
	IgG3	1.371	**0.041**	1.013	1.855	1.159	0.339	0.857	1.566
	IgM	1.605	**0.020**	1.057	2.438	1.148	0.522	0.753	1.749
	OPA	1.018	**0.004**	1.006	1.031	1.009	0.184	0.996	1.022
Pfs230	IgG1	1.547	0.552	0.368	6.504	1.056	0.933	0.296	3.761
	IgG3	1.449	0.332	0.685	3.067	1.005	0.990	0.496	2.035
	IgM	1.304	0.171	0.892	1.906	0.951	0.803	0.643	1.408
	OPA	1.043	**0.008**	1.010	1.076	1.024	0.117	0.994	1.055

N=261 children. OR: odds ratio; aOR: adjusted OR.

P values are two-sided and not adjusted for multiple comparisons

* Adjusted for age as a continuous variable, and household mosquito exposure as a categorical variable, 0-8, >8-40, >40-80, >80 mosquitos/household/day.

Supplementary Material

Supplementary Table S5: Relationship between Tfh subsets and odds of symptoms given infection

	Univariate				Adjusted			
**Tfh subset**	**OR**	**p**	**95% CI**		**aOR**	**p**	**95% CI**	
Tfh (% of CD4)	0.843	**0.017**	0.732	0.97	0.963	0.577	0.845	1.099
FoxP3 (% of Tfh)	1.116	0.388	0.87	1.432	1.116	0.324	0.897	1.39
Th1 (% of Tfh)	0.972	**0.005**	0.953	0.992	0.99	0.307	0.972	1.009
Th2 (% of Tfh)	1.028	**0.001**	1.011	1.045	1.012	0.124	0.997	1.029
Th17 (% of Tfh	0.979	0.401	0.932	1.029	0.975	0.251	0.933	1.018
ICOS (% of Tfh)	1.04	**0.026**	1.005	1.076	1.005	0.785	0.972	1.038
ICOS (% of Th1-Tfh)	1.019	0.091	0.997	1.041	0.996	0.684	0.975	1.017
ICOS (% of Th2-Tfh)	**1.014**	0.32	0.986	1.043	0.995	0.731	0.97	1.022
ICOS (% of Th17-Tfh)	1.026	**0.042**	1.001	1.052	1.009	0.406	0.987	1.032
Ki67 (% of Tfh)	1.088	0.053	0.999	1.185	1.085	**0.026**	1.01	1.165
Ki67 (% of Th1-Tfh)	1.041	0.212	0.977	1.11	1.014	0.654	0.955	1.076
Ki67 (% of Th2-Tfh)	1.078	0.077	0.992	1.172	1.091	**0.012**	1.019	1.167

N=207 children. OR: odds ratio; aOR: adjusted OR.

P values are two-sided and not adjusted for multiple comparisons

* Adjusted for age as a continuous variable

Supplementary Material

Supplementary Table S6: Relationship between antibodies and odds of symptoms given infection

			Univariate			Adjusted			
**Target**	**Type**	**OR**	**p**	**95% CI**		**aOR**	**p**	**95% CI**	
MSP2	IgG1	0.5032484	**<0.001**	0.39088	0.647915	1.101556	0.394	0.881945	1.375851
	IgG3	0.5032484	**<0.001**	0.39088	0.647915	0.7135221	**0.02**	0.536777	0.948465
	IgM	0.609609	**0.002**	0.44568	0.833835	0.8112374	0.169	0.602301	1.092654
	C1q	0.8766627	0.238	0.70453	1.090855	1.08813	0.412	0.889117	1.331687
	FcRIIa	0.8849889	0.264	0.71407	1.096814	1.125994	0.254	0.918337	1.380606
	FcRIII	0.8355886	0.08	0.68336	1.021721	1.071807	0.483	0.882897	1.301137
	OPA	0.9752543	**0.039**	0.95233	0.998733	0.9965761	0.763	0.974611	1.019036
AMA1	IgG1	0.7731162	**0.002**	0.65773	0.908746	1.000342	0.997	0.84742	1.180858
	IgG3	0.6401789	**0.009**	0.45722	0.896346	0.8272131	0.235	0.604828	1.131366
	IgM	0.3772019	**<0.001**	0.23231	0.612453	0.6424467	0.062	0.403874	1.021946
	C1q	0.8089297	0.089	0.63374	1.032542	1.072248	0.542	0.857082	1.341431
	FcRIIa	0.7500611	**<0.001**	0.64153	0.876951	0.9508174	0.536	0.810405	1.115558
	FcRIII	0.7197564	**0.001**	0.59268	0.874081	0.981101	0.851	0.803646	1.1977
	OPA	0.9782342	**0.001**	0.96538	0.991257	0.9940388	0.368	0.981186	1.00706
CSP	IgG1	0.5546815	**0.008**	0.35994	0.85478	0.7542221	0.144	0.516418	1.101533
	IgG3	0.8224371	0.183	0.61678	1.096675	1.030554	0.812	0.803895	1.32112
	IgM	0.6637743	**0.038**	0.45037	0.978302	1.037513	0.842	0.722009	1.490887
	OPA	0.9785899	**0.001**	0.96645	0.990885	0.9911169	0.136	0.979556	1.002815
Pfs230	IgG1	1.293855	0.71	0.3332	5.024239	1.602718	0.444	0.478933	5.363385
	IgG3	0.2195839	**0.019**	0.06157	0.783102	0.525503	0.193	0.199387	1.385014
	IgM	0.5397599	**0.003**	0.35879	0.812006	0.9051355	0.631	0.602777	1.359159
	OPA	0.9665171	**0.021**	0.93903	0.994805	0.9878649	0.346	0.963083	1.013285

N=255 children. OR: odds ratio; aOR: adjusted OR.

P values are two-sided and not adjusted for multiple comparisons

* Adjusted for age as a continuous variable.

Supplementary Material

Supplementary Table S7: Flow cytometry antibodies

**Target**	**Clone**	**Fluorophore**	**Supplier**	**Cat number**	**Dilution**
Malaria exposed Tfh phenotyping					
CD4	RPA-T4	PerCP Cy5.5	Biolegend	300530	1/50
CXCR5	J252D4	BV711	Biolegend	356934	1/83.33
PD-1	EH12.1	PE	BD Biosciences	560795	1/16.67
CXCR3	1C6	BV421	BD Biosciences	562558	1/31.25
CCR6	11A9	APC R700	BD Biosciences	565173	1/50
FoxP3	150D	Alexa 647	Biolegend	320014	1/83.33
ICOS	C398.4A	APC-Cy7	Biolegend	313530	1/62.5
Ki67	B56	FITC	BD Biosciences	556026	1/62.5
Live Dead		Aqua	Invitrogen	L34965	1/250
Malaria naïve Tfh phenotyping					
CD3	SK7	FITC	Biolegend	344804	1/10
CD4	OKT4	PerCP/Cyanine5.5	Biolegend	317428	1/250
CXCR5	J252D4	Brilliant Violet 711	Biolegend	356934	1/50
PD-1	EH12.1	PE-Cy7	BD Biosciences	561272	1/100
CXCR3	1C6	Brilliant Violet 421	BD Biosciences	562558	1/50
CCR6	11A9	Brilliant Violet 650	BD Biosciences	563922	1/100
Live Dead		Zombie NIR	Biolegend	423105	

Supplementary Material

Supplementary Figure S1

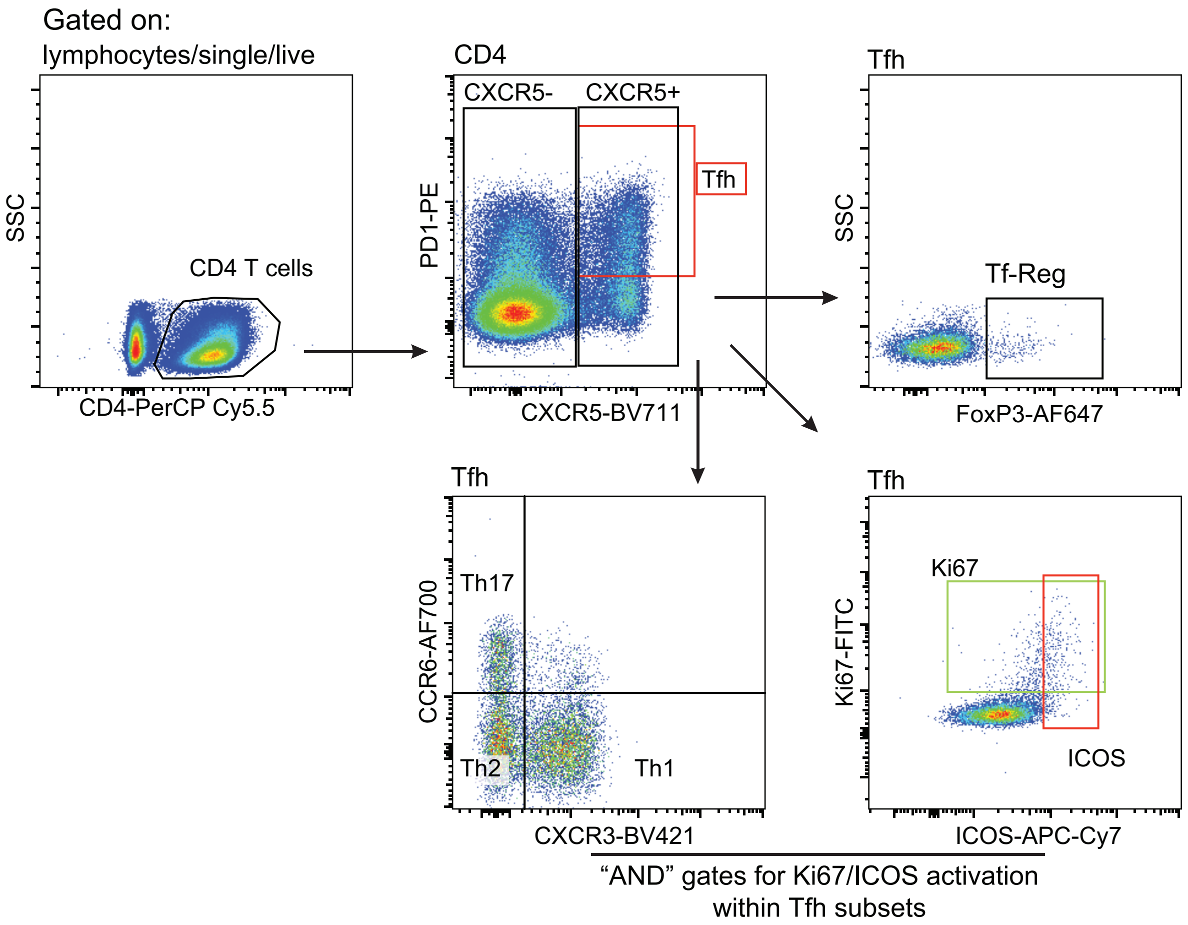

Supplementary Figure S1: Identification of Tfh

Gating strategy to identify cTfh, Tf-regulatory (Tf-Reg) cells, subsets and activation. CD4 T cells were gated as CD4+ from lymphocytes/single/live cells. Tfh cells were analysed based on PD1+CXCR5+ cells. Total CXCR5+ and CXCR5- cells were also analysed. Tf-regulatory cells (Tf-Reg) were identified by FoxP3 staining within Tfh cells. cTfh cell subsets were analysed based on CXCR3 and CCR6 staining into Th1 (CXCR3+CCR6-), Th17 (CXC3-CCR6+) and Th2 (CXCR3-CCR6-). Activation was gated as Ki67+ and ICOS+. "AND" gates were made to assess activation markers within subsets.

Supplementary Material

Supplementary Figure S2

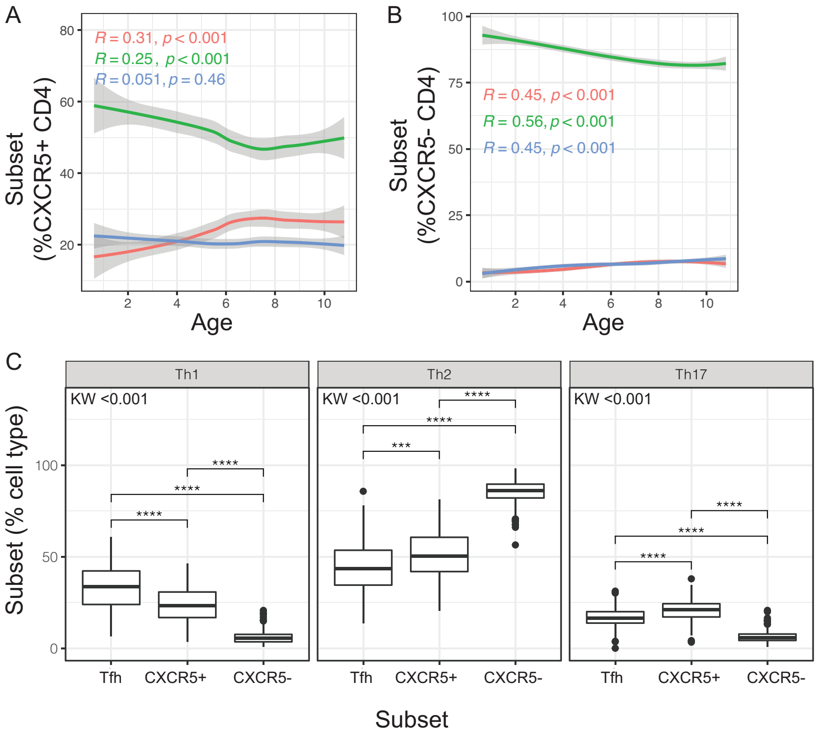

***Supplementary Figure S2: Subsets distribution in CXCR5+ and CXCR5- CD4 T cells. A/B)** CXCR5+ and CXCR5- CD4 T cells were analysed based on CXCR3 and CCR6 expression into Th1 (CXCR3+CCR6-), Th2 (CXCR3-CCR6-) and Th17 (CXCR3-CCR6+) subsets. The relationship of the proportion of each subsets within CXCR5+ (**A**) and CXCR5- cells (**B**) with age. Solid lines are LOESS fit curves with error bands of 95% confidence interval. Spearman rho and p are indicated. **C**) Subset distributions in Tfh, CXCR5+ and CXCR5- CD4 T cells were compared. Kruskal Wallis and Dunn post analysis FDR adjusted indicated. *** p<0.001, **** p< 0.0001. Box and whisker plots, box indicates first and third quartiles for hingers, median line, and whiskers are lowest and highest values no further than 1.5 interquartile range from hinges. Data beyond whisker lines are indicated with points and are treated as outliers. Data is from n=212 children. Source data are provided as a Source Data file.*

Supplementary Material

Supplementary Figure S3

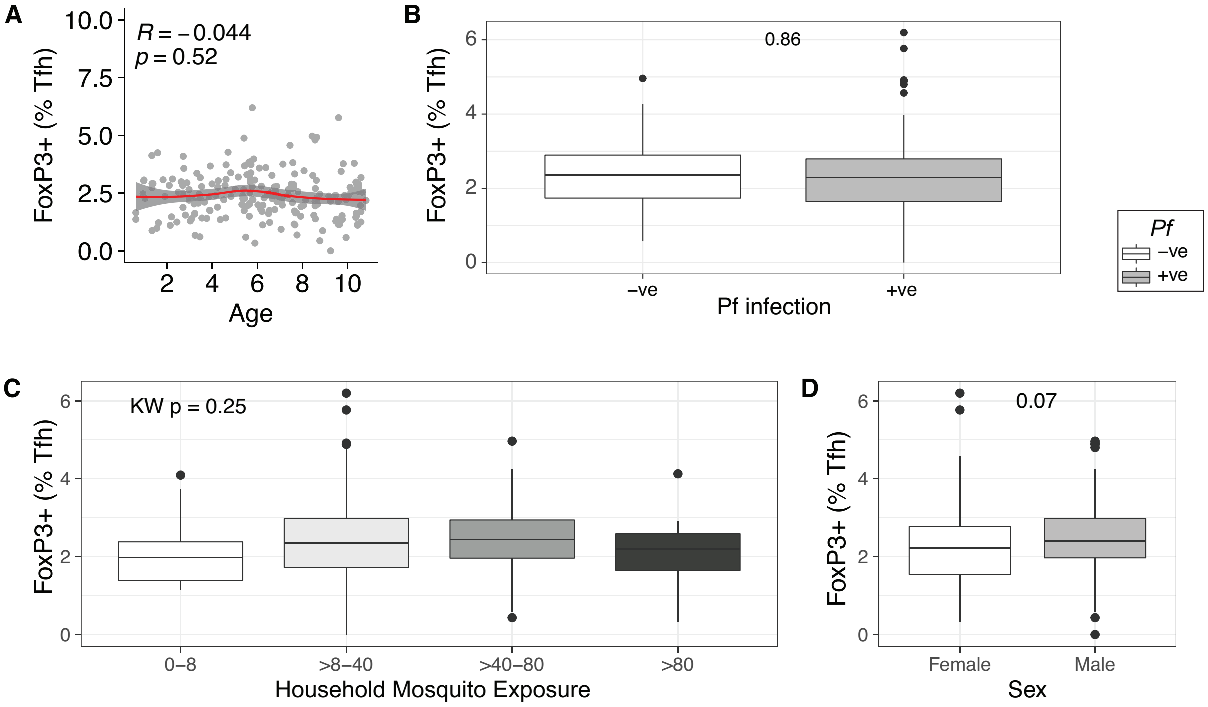

Supplementary Figure S3: Impact of age and infection on Tf-regulatory cells
Tf-regulatory cells were identified as FoxP3+ cells within the Tfh cell compartment. ***(A)*** *The relationship of the proportion of FoxP3 cells as proportion of Tfh cells with age. Line is LOESS fit curves with error bands of 95% confidence interval. Spearmans rho and P indicated.* ***(B)*** *FoxP3 cells and current asymptomatic P. falciparum infection. Mann Whitney U test indicated.* ***(C)*** *Household mosquito exposure (mean mosquitos/house/night). Kruskal Wallis indicated.* ***(D)*** *Sex. Mann Whitney U test indicated. Box and whisker plots, box indicates first and third quartiles for hingers, median line, and whiskers are lowest and highest values no further than 1.5 interquartile range from hinges. Data beyond whisker lines are indicated with points and are treated as outliers. Data is from n=212 children. All statistical tests are two-sided, with no adjustment for multiple comparisons. Source data are provided as a Source Data file.*

Supplementary Material

Supplementary Figure S4

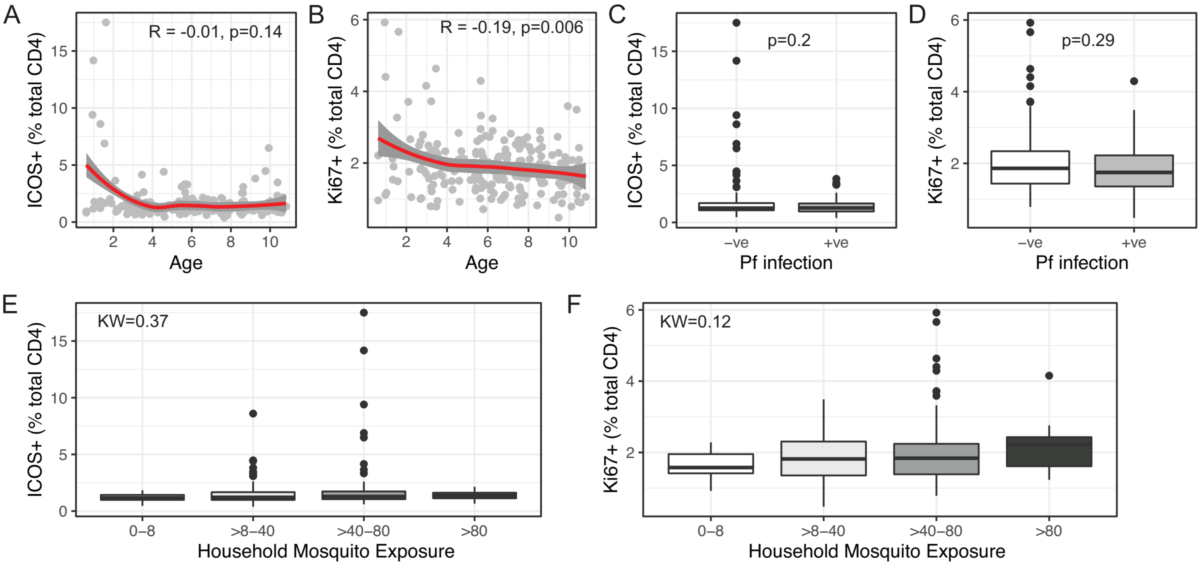

Supplementary Figure S4: Impact of age and malaria on total CD4 T cell activation and proliferation.
Activation and proliferation of total CD4 T cells was measured by ICOS and Ki67 expression. ***A/B)*** *The relationship between ICOS and Ki67 expression on total CD4 T cells with age. Line is LOESS fit curves with error bands of 95% confidence interval. Spearmans rho and p indicated.* ***C/D)*** *The relationship between ICOS and Ki67 expression on total CD4 T cells with current asymptomatic infection. Mann-Whitney U test indicated.* ***E/F)*** *The relationship between ICOS and Ki67 expression on total CD4 T cells and household mosquito exposure. Kruskal Wallis indicated. Box and whisker plots, box indicates first and third quartiles for hingers, median line, and whiskers are lowest and highest values no further than 1.5 interquartile range from hinges. Data beyond whisker lines are indicated with points and are treated as outliers. Data is from n=212 children. All statistical tests are two-sided, with no adjustment for multiple comparisons. Source data are provided as a Source Data file.*

Supplementary Material

Supplementary Figure S5

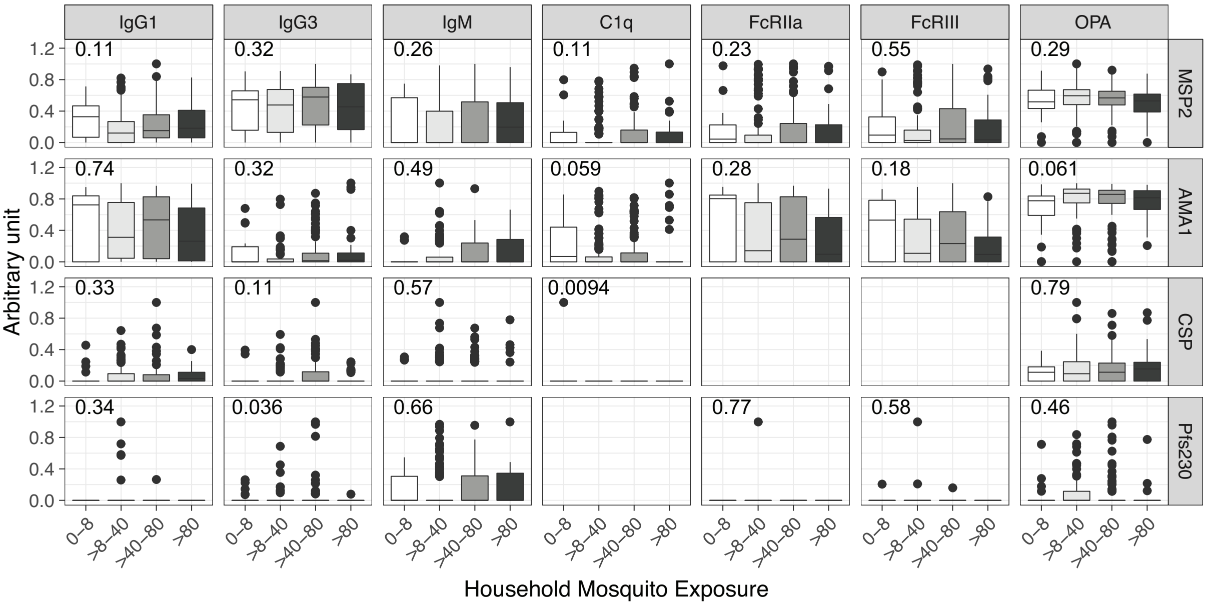

Supplementary Figure S5: Relationship between antibodies and household mosquito exposure

Magnitude of IgG1, IgG3, IgM and functional antibodies that mediated complement fixation (C1q), cross linked FcRIIa and FcRIII and mediated opsonic phagocytosis (OPA) to blood stage (MSP2, AMA1), sporozoite stage (CSP) and gametocyte (Pfs230) antigens with Household Mosquito Exposure (mean mosquitos/household/day). Antibody magnitude is expressed as arbitrary units, which are calculated by thresholding data at positive seroprevalence levels and scaling data to highest responder. Kruskal Wallis P value is indicated. Data is from n=262 children. . Box and whisker plots, box indicates first and third quartiles for hingers, median line, and whiskers are lowest and highest values no further than 1.5 interquartile range from hinges. Data beyond whisker lines are indicated with points and are treated as outliers. Source data are provided as a Source Data file.

Supplementary Material

Supplementary Figure S6

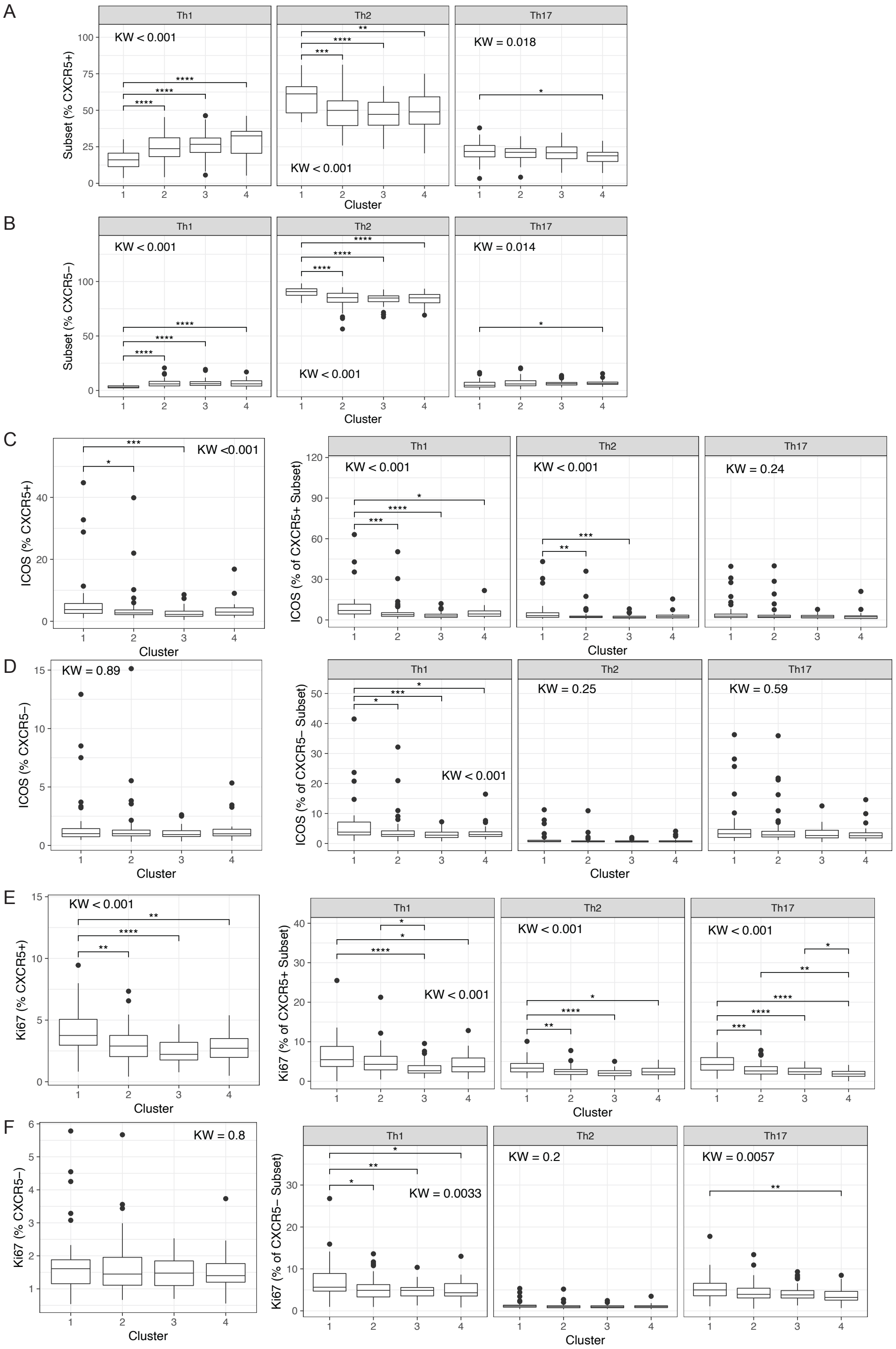

Supplementary Figure S6: Relationships between clusters and CXCR5- and CXCR5+ CD4 T cells.

Tfh and antibody responses were analysed by PCA and individuals clustered by kmeans. ***A/B)*** *Subset distribution of CXCR5+ and CXCR5- CD4 cells in kmeans clusters.* ***C-F)*** *ICOS and Ki7 on total and subsets of CXCR5- and CXCR5+ CD4 T cells in kmeans clusters. Kruskal Wallis and Dunn post analysis FDR adjusted indicated. * p<0.05, **p<0.01, ***p<0.001, ****p<0.0001. Data is from n=212 children. Box and whisker plots, box indicates first and third quartiles for hingers, median line, and whiskers are lowest and highest values no further than 1.5 interquartile range from hinges. Data beyond whisker lines are indicated with points and are treated as outliers. Source data are provided as a Source Data file.*
