# Supplementary material for: Age-dependent changes in circulating Tfh cells influence development of functional malaria antibodies in children
Source: Nat Commun. 2022 Jul 18;13:4159. doi: 10.1038/s41467-022-31880-6 (PMC9293980; doi:10.1038/s41467-022-31880-6)
Supplement: Supplementary file 3 — Reporting Summary [file 41467_2022_31880_MOESM3_ESM.pdf]

nature portfolio

Corresponding author(s): Michelle Boyle

Last updated by author(s): May 12, 2022

Reporting Summary

Nature Portfolio wishes to improve the reproducibility of the work that we publish. This form provides structure for consistency and transparency in reporting. For further information on Nature Portfolio policies, see our Editorial Policies and the Editorial Policy Checklist.

Statistics

For all statistical analyses, confirm that the following items are present in the figure legend, table legend, main text, or Methods section.

n/a	Confirmed	
☐	☒	The exact sample size (*n*) for each experimental group/condition, given as a discrete number and unit of measurement
☐	☒	A statement on whether measurements were taken from distinct samples or whether the same sample was measured repeatedly
☐	☒	The statistical test(s) used AND whether they are one- or two-sided<br>*Only common tests should be described solely by name; describe more complex techniques in the Methods section.*
☐	☒	A description of all covariates tested
☐	☒	A description of any assumptions or corrections, such as tests of normality and adjustment for multiple comparisons
☐	☒	A full description of the statistical parameters including central tendency (e.g. means) or other basic estimates (e.g. regression coefficient) AND variation (e.g. standard deviation) or associated estimates of uncertainty (e.g. confidence intervals)
☐	☒	For null hypothesis testing, the test statistic (e.g. *F*, *t*, *r*) with confidence intervals, effect sizes, degrees of freedom and *P* value noted<br>*Give P values as exact values whenever suitable.*
☒	☐	For Bayesian analysis, information on the choice of priors and Markov chain Monte Carlo settings
☒	☐	For hierarchical and complex designs, identification of the appropriate level for tests and full reporting of outcomes
☐	☒	Estimates of effect sizes (e.g. Cohen's *d*, Pearson's *r*), indicating how they were calculated

Our web collection on statistics for biologists contains articles on many of the points above.

Software and code

Policy information about availability of computer code

Data collection	No custom software was used.
Data analysis	Flow cytometry analysis was performed in FLowJo (version 10). All statistical data analyses were performed using Prism 7 (GraphPad version 7.0d), STATA (version 15) and RStudio (R version 4.0.4). All statistical tests are two sided. For analysis of antibody levels, antibody magnitude is expressed as arbitrary units, which are calculated by thresholding data at positive seroprevalence levels and scaling data to highest responder. Correlations between antibody variables and age were assessed using Pearson correlation for continuous variables. Clustering of correlations was performed with H-clustering with Ward.D method with R Hmisc package (version 4.7-0). To assess the association between Tfh cells and antibodies once controlling for age, lower limit of antibodies magnitudes were set at 0.001 and then log transformed before analysis in linear regression modelling. For clustering analysis of individuals, only those with complete Tfh and antibody data were included. All data was transformed into z-scores and then PCA performed in factoextra (version 1.0.7) and FactoMineR (version 2.4). Kmeans cluster number chosen in 'useful' package (version 1.2.6) and kmeans clusters identified with 'stats' package (version 4.1.2) in R . Four clusters were chosen based on Hartigan's rule with an inclusion value of ~10, and sample size consideration. For LASSO Poisson regression modeling was used to select the most informative prognostic factors for 1) incidence of density infections and 2) incidence of symptoms when infected across the multiple visits during the study period. Variables assessed include age, gender, daily mosquito exposure rate (continuous in log and categorical 0-8,>8-40, >40-80, >80), and OD values from 27 antibodies: The optimum lambdas were selected using 5-fold cross validation that achieves the minimum Poisson deviance. Relative variable importance of selected variables was described using standardized coefficients. Only children with complete information were included in the analyses (complete case analyses, n=211). R statistical software version 4.0.2 with R package 'glmnet' (version 4.1.4) was used

For manuscripts utilizing custom algorithms or software that are central to the research but not yet described in published literature, software must be made available to editors and reviewers. We strongly encourage code deposition in a community repository (e.g. GitHub). See the Nature Portfolio guidelines for submitting code & software for further information.

Data

Policy information about availability of data

All manuscripts must include a data availability statement. This statement should provide the following information, where applicable:

- Accession codes, unique identifiers, or web links for publicly available datasets
- A description of any restrictions on data availability
- For clinical datasets or third party data, please ensure that the statement adheres to our policy

All data generated or analysed during this study are included in this published article (and its supplementary information files). Source data are provided as a Source Data file.

Field-specific reporting

Please select the one below that is the best fit for your research. If you are not sure, read the appropriate sections before making your selection.

[x] Life sciences [ ] Behavioural & social sciences [ ] Ecological, evolutionary & environmental sciences

For a reference copy of the document with all sections, see nature.com/documents/nr-reporting-summary-flat.pdf

Life sciences study design

All studies must disclose on these points even when the disclosure is negative.

Sample size	No sample size calculations were performed. All available PBMC and plasma samples for clinical cohorts were used.
Data exclusions	No data was excluded from analysis
Replication	All antibody measures were preformed in duplicate, and repeated if replicates had >25% difference. For flow cytometry analysis, replicates for individual were not possible. Assays were optimised on healthy donors prior to analysis of clinical samples, with repeated analysis of healthy donors to confirm reproducibility of results.
Randomization	No randomization was performed in this study. All plasma/PBMC samples from clinical cohorts were analysed.
Blinding	Investigators were blinded to clinical/demographic data during antibody and flow cytometry data generation and analysis. Post analysis, data was matched with clinical and demographic data.

Reporting for specific materials, systems and methods

We require information from authors about some types of materials, experimental systems and methods used in many studies. Here, indicate whether each material, system or method listed is relevant to your study. If you are not sure if a list item applies to your research, read the appropriate section before selecting a response.

Materials & experimental systems

n/a	Involved in the study	
[ ]	[x]	Antibodies
[ ]	[x]	Eukaryotic cell lines
[x]	[ ]	Palaeontology and archaeology
[x]	[ ]	Animals and other organisms
[ ]	[x]	Human research participants
[x]	[ ]	Clinical data
[x]	[ ]	Dual use research of concern

Methods

n/a	Involved in the study	
[x]	[ ]	ChIP-seq
[ ]	[x]	Flow cytometry
[x]	[ ]	MRI-based neuroimaging

Antibodies

Antibodies used

All details of antibodies are in Supplementary Table S7

Target Clone Fluorophore Supplier Cat number
Malaria exposed Tfh phenotyping
CD4 RPA-T4 PerCP Cy5.5 Biolegend 300530 1/50 dilution
CXCR5 J252D4 BV711 Biolegend 356934 1/83.3 dilution
PD-1 EH12.1 PE BD Biosciences 560795 1/16.7 dilution
CXCR3 1C6 BV421 BD Biosciences 562558 1/31.25 dilution

CCR6 11A9 APC R700 BD Biosciences 565173 1/50 dilution
ICOS C398.4A APC-Cy7 Biolegend 313530 1/62.5 dilution
Ki67 B56 FITC BD Biosciences 556026 1/62.5 dilution
FoxP3 150D AF647 Biolegend 320014 1/83.33
Live Dead Aqua Invitrogen L34965 1/250

Malaria naïve Tfh phenotyping
CD3 SK7 FITC Biolegend 344804 1/10 dilution
CD4 OKT4 PerCP/Cyanine5.5 Biolegend 317428 1/250 dilution
CXCR5 J252D4 Brilliant Violet 711 Biolegend 356934 1/50 dilution
PD-1 EH12.1 PE-Cy7 BD Biosciences 561272 1/100 dilution
CXCR3 1C6 Brilliant Violet 421 BD Biosciences 562558 1/50 dilution
CCR6 11A9 Brilliant Violet 650 BD Biosciences 563922 1/100 dilution
Live Dead Zombie NIR Biolegend 423105 13/20000 dilution

ELISA antibodies
goat anti-human IgG HRP-conjugated ThermoFisher Scientific cat#2-8420, 1/1000 dilution
mouse anti-human IgG1 clone HP6069 Thermo Fisher Scientific cat# A-10630, 1/1000 dilution
mouse anti-human IgG2 clone HP6002 Thermo Fisher Scientific cat# 05-3500, 1/1000 dilution
mouse anti-human IgG3 clone HP6050 Thermo Fisher Scientific cat# 05-3600 dilution
mouse anti-human IgG4 clone HP6025 Thermo Fisher Scientific cat# A-10651, 1/1000 dilution
mouse anti-human IgM clone HP6083 Thermo Fisher Scientific cat# 054900 1/1000 dilution
goat anti-mouse HRP conjugated Millipore cat# AP308P, 1/1000 dilution

Validation

Antibody validations were performed by the suppliers per quality assurance documents provided by each supplier. All validation data are available on the manufacturer's websites.

Eukaryotic cell lines

Policy information about cell lines

Cell line source(s)

THP-1 monocyte cell lines were obtained from ATCC

Authentication

THP-1 cells were not independently validated, but Fc-gamma-receptor expression was confirmed by Flow Cytometry.

Mycoplasma contamination

THP-1 cells were not tested for mycoplasma contamination.

Commonly misidentified lines
(See ICLAC register)

NA

Human research participants

Policy information about studies involving human research participants

Population characteristics	Participant characteristics collected include age, sex, and for Ugandan cohort, current, and future malaria infection. This information is available in Supplementary Table 1.<br><br>N= 262<br>age (median IQR) 5.86 (3.7-8.3)<br>female n=117 45%<br>Pf infection at blood draw n=106, n40%<br>Household mosquito exposure 0-8 (n=21, 8%), >8-40 (n==124, 47%), >40-80 (n=91, 35%), >80 (n=26, 10%)<br>Any infection in the year following n=241 (92%)
Recruitment	Ugandan cohort:<br>Samples were obtained from 262 children (117 female/145male) enrolled in a longitudinal study by the East African International Centres of Excellence in Malaria Research conducted in the high transmission areas of Uganda 29,31,67. This cohort consists of 100 households within the rural Nagongera sub-county in the Tororo district, where malaria transmission is holoendemic with seasonal peaks from October to January and April to July. All households within the subcounties were enumerated and mapped using handheld global positioning systems (Garmin e-Trex 10 GPS unit, Garmin International Inc., Olathe, KS). A household was defined as any single permanent or semipermanent dwelling acting as the primary residence for a person or group of people that generally cook and eat together. Using a computerized number generator, random samples of households from each subcounty were approached consecutively, and 100 households were enrolled per site into both the entomologic surveys and cohort studies if they met the following criteria: 1) at least one household resident 0.5–10 years of age and 2) at least one adult resident available for providing informed consent. As such, no-self selection bias is involved in this study, and other bias in recruitment are limited. Following selection of the household, one adult caregiver (>20 years) and all children (eligibility of those aged 6 months to 10 years) were enrolled in the study. Upon enrollment all study participants were given an insecticide treated bed net and followed for all medical care at a dedicated study clinic. Children who presented with a fever (tympanic temperature >38.0 °C) or history of fever in the previous 24 hours had blood obtained by finger prick for a thick smear. If the thick smear was positive for Plasmodium parasites, the patient was diagnosed with malaria regardless of parasite density, and treated with artemether-lumefantrine. Routine assessments were performed in the study clinic every three months, including blood smears (which were assessed for both blood stage and gametocyte parasites) and dry blood spots to detect for parasite infection by PCR. Negative blood smears obtained at routine assessments were tested for the presence of submicroscopic malaria parasites using loop-mediated isothermal amplification (LAMP). Blood was drawn from each participant at a single cross-sectional timepoint between January and April 2013. Participant demographics for the current study are in Supplementary Table S1. Participants were reimbursed travel costs for all visits to study clinic.<br><br>Malaria naïve cohort: PBMCs were collected from a healthy malaria-naive cohort of children (n=13, median age 8 IQR [3-13], 38% female) and adults (n=14, median age 39.5 IQR [25-43], 43% female) from a clinic of hospital outpatients. Volunteers were assessed by an on-site immunologist, where they were confirmed immunologically healthy and malaria-naïve. No compensation was provided for participants in this cohort.
Ethics oversight	Ethics approval for the use of human samples was obtained from the Makeree Univestiy School of Medicine Research and Ethics Committee (2011-167), the Uganda National Council of Science and Technology (HS1019), the University of California, San Francisco Committee of Human Research (11-05995), and the Alfred Health Ethics Committee (#328/17), QIMR-Berghofer Human Research Ethics Committee (P3444 and P3445), Stanford University Institutional Review Board (IRB 41197) and Menzies School of Health Research Human Ethics Research Committees (2012-1766). Written informed consent was obtained from all adult study participants and parents or legal guardians of the children.

Note that full information on the approval of the study protocol must also be provided in the manuscript.

Flow Cytometry

Plots

Confirm that:

- ☒ The axis labels state the marker and fluorochrome used (e.g. CD4-FITC).
- ☒ The axis scales are clearly visible. Include numbers along axes only for bottom left plot of group (a 'group' is an analysis of identical markers).
- ☒ All plots are contour plots with outliers or pseudocolor plots.
- ☒ A numerical value for number of cells or percentage (with statistics) is provided.

Methodology

Sample preparation	Ex vivo Tfh phenotype and activation was assessed by flow cytometry. PBMCs were thawed in 10% FBS/RPMI, and rested for 2 hours at 37 oC,5% CO2. In brief, 1M PBMCs were stained with surface antibodies to identify Tfh subsets, CD3, CD4, CXCR5 PD-1, CXCR3 and CCR6, activation markers included ICOS (Supplementary Table S7). PBMCs were stained for 15 mins at RT, washed with 2% FBS/PBS, for intracellular markers, PBMCs were permeabilised with CytoFix/CytoPerm (BD) and 1 X Perm/

	Wash (BD) and stained with intracellular markers Ki67 and FoxP3.
Instrument	Samples were acquired on Aurora Cytek 3 laser instrument (Australian samples) or an Attune NXT Flow cytometer (Ugandan samples).
Software	Data was analysed in FlowJo v 10.
Cell population abundance	No cell sorting was performed in this study.
Gating strategy	Gating strategy is outlined in Supplementary Figure S1. Single cells were gated on FSC-H/FSC-A and SSC-H/SSC-A and then intact lymphocytes were gated on FSC-A/SCC-A and then live cells gated as Aqua or Zombie NIR negative. Single/lymphocyte/live cells were gated as CD4 and then analysed as outlined in Supplementary Figure S1.

☒ Tick this box to confirm that a figure exemplifying the gating strategy is provided in the Supplementary Information.
